# PubMed-Indexed Productivity of Matched Orthopedic Surgery Applicants Before and After Step 1 Scoring Transition

**DOI:** 10.7759/cureus.90388

**Published:** 2025-08-18

**Authors:** Yagni Patel, Akshar Patel, Arjun Bhatt, Abdullah B Chandasir, Thomas Drake, Ali Abolhassani, Jackson McClain, Asim Ahmed, Uzondu Agochukwu

**Affiliations:** 1 Orthopedic Surgery, Medical College of Georgia at Augusta University, Augusta, USA

**Keywords:** orthopedic surgery, publications, pubmed, research productivity, residency

## Abstract

Introduction: While research productivity surrounding the Step 1 scoring transition has been assessed, the specific impact on verified PubMed-indexed publications (PMIDs) has not been assessed. No study has quantified what proportion of self-reported research items reported in National Resident Match Program (NRMP) data is actually PubMed-indexed. Addressing these gaps is essential to understanding how research output is evolving and represented in residency applications.

Objectives: The objective of this study is to evaluate how the Step 1 pass/fail transition affected pre-residency research output among matched orthopedic surgery residents and whether medical school National Institutes of Health (NIH) funding independently predicts research productivity.

Methods: This retrospective cohort study included 1,441 matched orthopedic surgery residents across two cycles: pre-transition (class of 2026) and post-transition (class of 2029). PubMed was used to identify total, first-author, in-specialty publications, and citation rates. Residents were categorized by medical school NIH funding and program tier. Mann-Whitney U tests compared groups, and negative binomial regression identified independent predictors.

Results: Research productivity increased significantly following the Step 1 transition. Post-transition residents published nearly twice as many PubMed-indexed articles as their pre-transition peers (IRR = 2.13, p < 0.001), with similar gains in first-author and in-specialty work. NIH funding and program tier were independent predictors across all metrics. Citation rates did not differ. Only 12-15% of reported abstracts, presentations, and publications (APPs) were PMIDs.

Conclusion: This study provides the first validated analysis of PubMed-indexed research output before and after the Step 1 transition. These findings can inform future studies across specialties as research output becomes increasingly central to residency selection.

## Introduction

Orthopedic surgery is among the most competitive specialties in the United States, with a consistent 100% fill rate since 2019 and an overall match rate of 61% in 2024 [1]. The United States Medical Licensing Examination (USMLE) is a three‑step examination process comprising Step 1, Step 2, and Step 3 that medical students and graduates must pass to obtain a medical license in the United States [2]. Historically, residency programs have relied on the United States Medical Licensing Exam (USMLE) Step 1 scores to screen applicants, with over 80% of orthopedic program directors (PDs) using it to reduce application volume [3]. However, the transition to pass/fail scoring of Step 1 in 2022 eliminated this objective filter, prompting an emphasis towards other application components, including USMLE Step 2 scores, clinical grades, and research activity. Recent literature and national survey data indicate that research productivity has become a more prominent selection criterion in the wake of the grading change to Step 1, especially in competitive specialties where applicant volume and academic expectations remain high [4,5].

Previous studies have described rising research output among orthopedic surgery applicants, with recent findings from Lin et al. reporting a statistically significant rise in total abstracts, presentations, and publications (APPs) from the 2011 to 2024 National Resident Match Program (NRMP) Charting Outcomes data [6]. However, no study to date has quantified changes specifically in PubMed-indexed publications (PMIDs) before and after the Step 1 transition as opposed to the above-stated rise in APPs. This distinction is important, as PMIDs represent a higher standard for scholarly output than conference abstracts, posters, or presentations [7]. Moreover, while students from highly National Institutes of Health (NIH)-funded medical schools are believed to have greater access to research mentorship and infrastructure, few studies have examined how institutional funding levels influence publication volume among matched orthopedic surgery residents [8]. As research productivity becomes a more emphasized and potentially differentiating metric in residency selection, understanding how recent policy shifts and institutional factors influence publication output is essential.

The goals of this study therefore are twofold: (1) to contextualize pre-residency research productivity of matched orthopedic surgery residents to the mean APPs published by the NRMP before and after the Step 1 transition and (2) to assess the association between the medical school NIH funding level and publication output. By analyzing a national cohort of orthopedic residents with standardized PMID data, this study offers a focused view of how recent policy changes and institutional context have shaped the research landscape for orthopedic surgery applicants.

## Materials and methods

Program selection

Using the Doximity Residency Navigator, all 207 orthopedic residency programs, ranked by “Reputation,” were analyzed in this study [9]. This platform was selected based on its use in prior studies with similar objectives [10,11]. The program rankings were only used for sorting purposes and were not factored into any analysis of data. Only programs with publicly available data on resident full names, degree types (MD, DO, or MD/PhD), and medical schools were included. Programs that did not meet these criteria were excluded and replaced by the next eligible program in the ranking. Overall, a total of 153 programs (73.9%) were included in the study, and 54 programs (26.1%) were excluded.

Resident selection

Residents from the classes of 2026 and 2029 were selected to capture cohorts before and after the Step 1 scoring transition to pass/fail. Given that medical students typically sit for the Step 1 exam after the summer of their second year, class of 2026 residents were presumed to have taken Step 1 in the summer of 2019 before the Step 1 transition, and class of 2029 residents were presumed to have taken Step 1 in the summer of 2022 after the Step 1 transition. MD/PhD residents were excluded due to the expected impact of dedicated research years on publication output [12].

Pre-residency research output collection

Mean APP values were obtained from the 2021 and 2024 NRMP Charting Outcomes data, respectively, for each class of residents analyzed in the study. PubMed was used to identify each resident’s total publications, first-author publications, and in-specialty (orthopedic-related) publications, using full name and affiliated medical school and/or hospital [13]. Searches were filtered to include only articles published before medical school graduation (≤May 1, 2021 for the class of 2026, ≤May 1, 2024 for the class of 2029). Each publication was manually determined as in-specialty or not based on the article title, content, and relevance to any orthopedic surgical procedure, diagnosis, pathology, education, diagnostics, treatments, techniques, or procedures. Citation counts for each publication were obtained and adjusted based on the number of years since publication to determine citation rates. For analytical purposes, publications were grouped into three categories: Original Research (encompassing journal articles, clinical trials, observational studies, and other forms of primary research), Reviews (including traditional reviews, systematic reviews, and meta-analyses), and Case Reports. To ensure the analysis focused on significant scholarly work, items such as preprints, letters to the editor, errata, corrections, and commentaries were excluded.

Medical school NIH funding classification

Medical school NIH funding levels were sourced from the 2024 Blue Ridge Institute for Medical Research (BRIMR) rankings, which listed 144 U.S. MD-granting institutions [14]. Schools were grouped into tiers: “High NIH Funding” (ranks 1-48), “Moderate NIH Funding” (ranks 49-96), “Low NIH Funding” (ranks 97-144). Residents were categorized based on the NIH funding tier of their medical school. Schools not included in the BRIMR list were labeled “Unknown NIH Funding.” 

Statistical analysis

Descriptive statistics were calculated for each outcome variable, and Mann-Whitney U tests were used to assess group differences due to non-normal data distribution. Statistical significance was defined as p < 0.05. To evaluate independent predictors of research productivity, we employed negative binomial regression models for total publications, first-author publications, and in-specialty publications. This model was selected to account for overdispersion in count data. Covariates included Step 1 transition status (pre vs. post), medical school NIH funding level (high vs. non-high), and residency program tier. Incidence rate ratios (IRRs) and Akaike Information Criterion (AIC) values were reported for each outcome. All analyses were conducted using Python (version 3.12).

## Results

A total of 1,441 matched orthopedic surgery residents were analyzed, with 727 residents in the pre-Step 1 transition group (class of 2026) and 714 residents in the post-Step 1 transition group (class of 2029). Approximately 39.2% of included residents graduated from medical schools with high NIH funding, followed closely by 31.2% from moderate NIH funding, 19.7% from low NIH funding, and 13.8% from programs with unknown NIH funding (Table 1).

**Table 1:** Summary of Residents by Step 1 Transition Phase Counts of matched orthopedic surgery residents included in the study, stratified by medical school NIH funding tier and Step 1 transition status (pre- vs. post-pass/fail). Funding tiers were derived from 2024 BRIMR rankings of U.S. MD-granting institutions. NIH: National Institutes of Health

	Pre-transition (n)	Post-transition (n)
High NIH Funding	270	296
Moderate NIH Funding	231	219
Low NIH Funding	112	113
Unknown NIH Funding	114	86
Total	727	714

**Figure 1:**
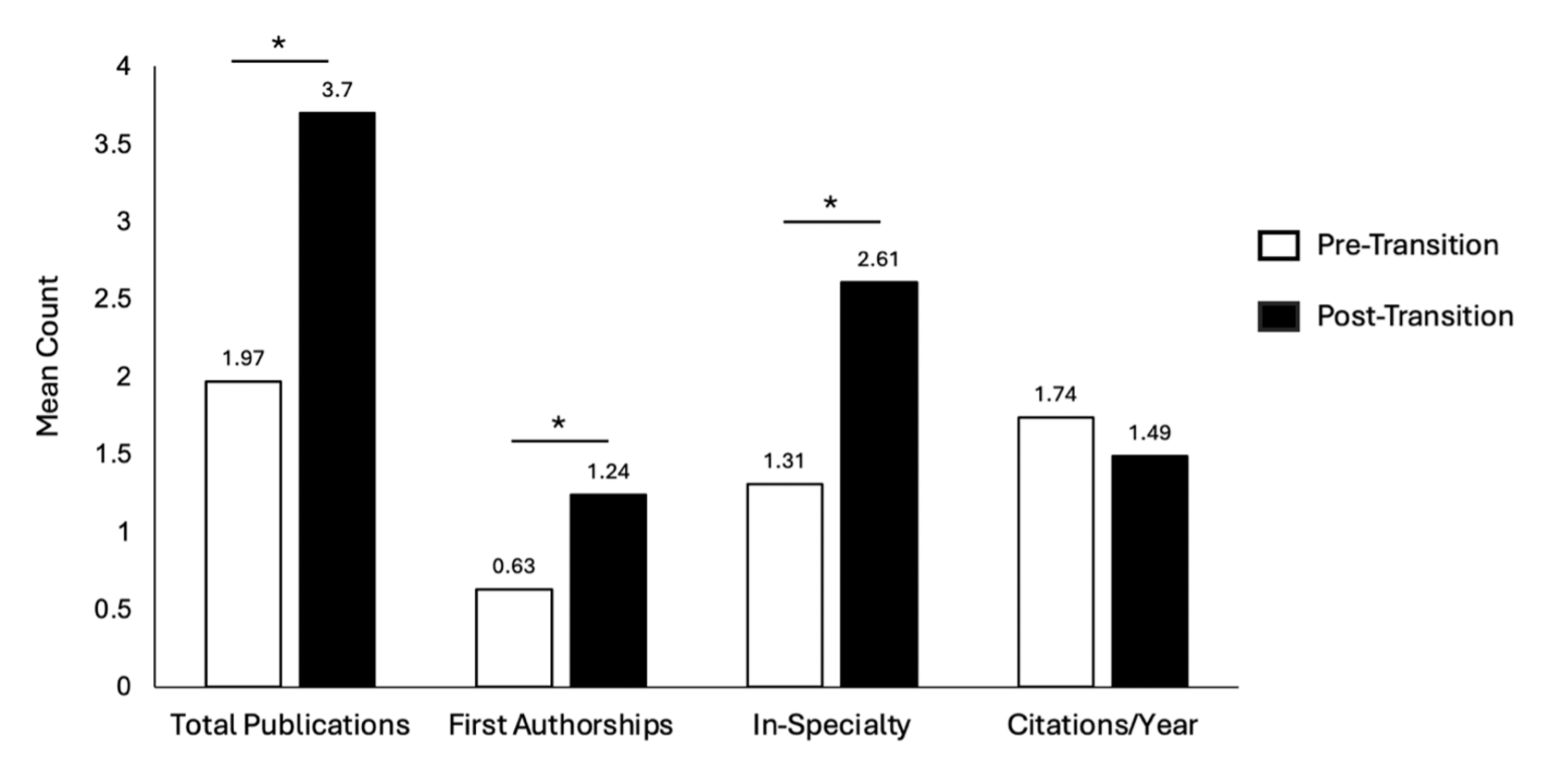
Research Output by Transition Phase Mean number of PubMed-indexed total publications, first-author publications, in-specialty publications, and normalized citations per year among orthopedic surgery residents in the pre-Step 1 (numeric score) and post-Step 1 (pass/fail) cohorts. Asterisks (*) denote statistically significant differences between groups (p < 0.05). All values represent verified publication metrics extracted from PubMed prior to residency start.

Post-transition residents (class of 2029) demonstrated significantly higher mean research productivity than pre-transition residents (class of 2026) across all major metrics (Figure 1). Total PMIDs increased from 1.97 ± 3.94 to 3.70 ± 6.24 (p < 0.001), first-author publications from 0.63 ± 1.77 to 1.24 ± 3.30 (p < 0.001), and in-specialty publications from 1.31 ± 3.27 to 2.61 ± 5.28 (p < 0.001). Normalized citation rates showed no significant change (1.74 vs. 1.49 citations/year; p = 0.72).

When stratified by the NIH funding level (Table 2), all three tiers-high, moderate, and low-showed statistically significant increases in total, first-author, and in-specialty publications following the Step 1 transition (all p < 0.01). However, citation rates did not significantly differ across funding tiers.

**Table 2:** Research Productivity Comparison by Step 1 Status Comparison of mean total publications, first-author publications, in-specialty publications, and citation counts between pre- and post–Step 1 transition cohorts across different medical school NIH funding tiers. All values reflect PubMed-indexed publications. Citation rates were normalized per year. Statistical significance was assessed using Mann-Whitney U tests. NIH: National Institutes of Health

	NIH Level	Pre-transition	Post-transition	p
Total Publications	High	3.10 ¬± 5.25	4.85 ¬± 6.49	<0.001
	Moderate	1.51 ¬± 2.81	3.70 ¬± 7.13	<0.001
	Low	1.06 ¬± 1.90	2.49 ¬± 3.59	<0.001
First Authorships	High	1.02 ¬± 2.50	1.54 ¬± 2.62	<0.001
	Moderate	0.45 ¬± 1.04	1.15 ¬± 2.31	<0.001
	Low	0.35 ¬± 0.75	0.85 ¬± 1.65	0.006
In-Specialty	High	2.10 ¬± 4.45	3.40 ¬± 5.49	<0.001
	Moderate	1.07 ¬± 2.43	2.85 ¬± 6.58	<0.001
	Low	0.55 ¬± 1.04	1.69 ¬± 2.93	<0.001
Citation Count	High	2.44 ¬± 3.96	2.10 ¬± 4.21	0.08
	Moderate	1.41 ¬± 2.57	1.29 ¬± 1.56	0.128
	Low	1.05 ¬± 2.28	1.25 ¬± 2.89	0.095

Negative binomial regression analyses confirmed that post-Step 1 transition status was independently associated with increased research output across all outcome domains. Compared to pre-transition applicants, post-transition applicants had significantly higher total publication counts (IRR = 2.13, p < 0.001), first-author publications (IRR = 2.28, p < 0.001), and in-specialty publications (IRR = 2.32, p < 0.001), even after adjusting for medical school NIH funding level and residency program tier. NIH funding and program tier were also significant independent predictors across all three models (Table 3).

**Table 3:** Negative Binomial Regression of Predictors of Research Productivity Results from negative binomial regression models assessing the independent effect of Step 1 transition status, medical school NIH funding level, and program tier (Doximity reputation rank) on total, first-author (1A), and in-specialty (IS) PubMed-indexed publication counts. Incidence rate ratios (IRRs) represent the multiplicative change in publication count associated with each predictor. NIH: National Institutes of Health

Outcome	Post-T IRR	p	High NIH IRR	p	Top Tier IRR	p	AIC
Total publications	2.134	<0.001	1.792	<0.001	1.748	<0.001	5982.2
1A publications	2.276	<0.001	1.746	<0.001	1.645	<0.001	3580.7
IS publications	2.319	<0.001	1.788	<0.001	1.729	<0.001	5000.9

**Figure 2:**
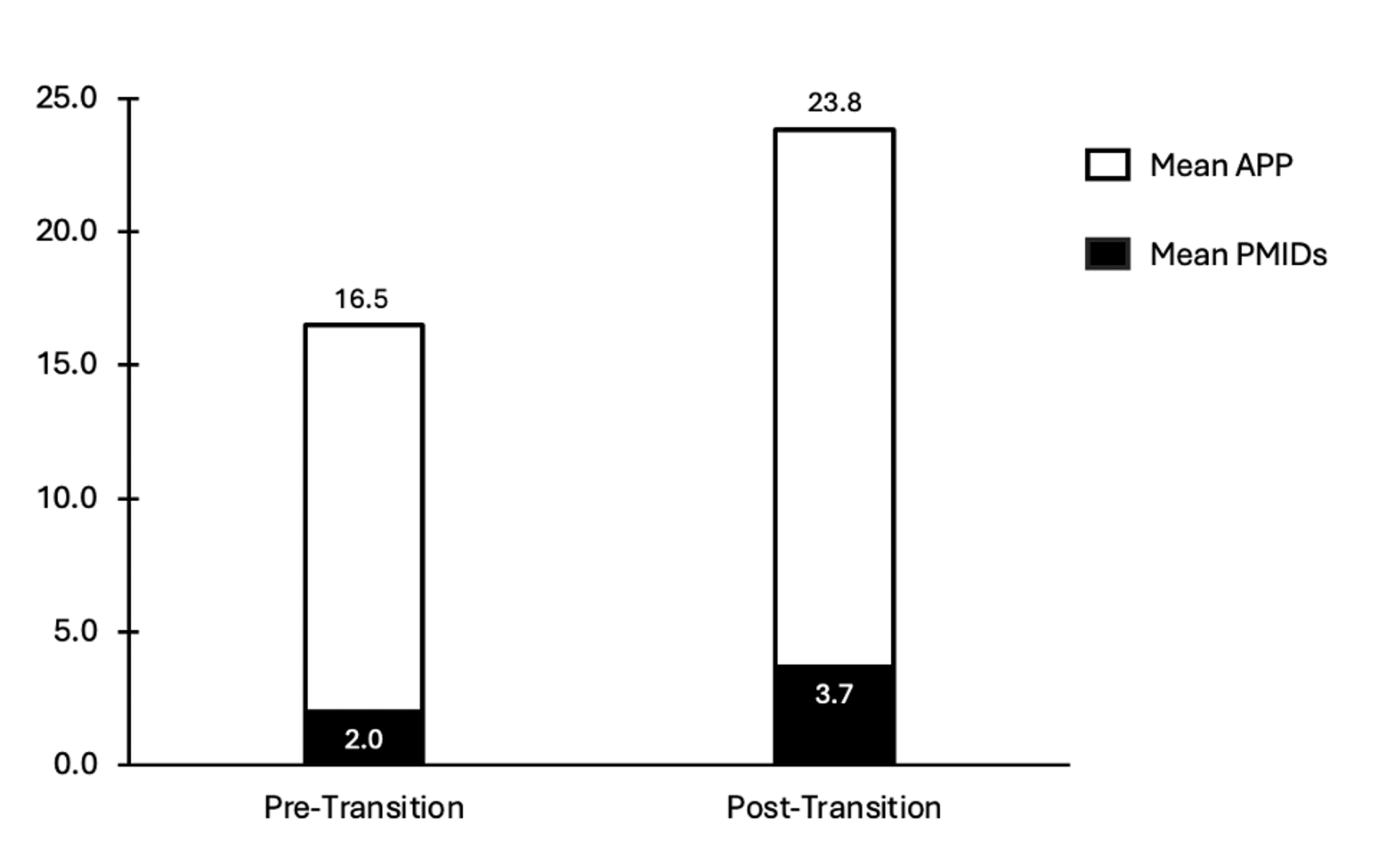
Estimated Proportion of PubMed-Indexed Publications Among Reported APPs Estimated proportion of total research items (APPs) that were PubMed-indexed publications (PMIDs) among matched orthopedic surgery applicants in the pre- and post–Step 1 transition cohorts. APP values were obtained from National Resident Match Program (NRMP) Charting Outcomes; PMID counts were extracted via structured search. This figure highlights a potential disconnect between rising APP totals and growth in peer-reviewed, indexed scholarship.

Despite increased research productivity in absolute terms, the proportion of self-reported research items that were PubMed-indexed remained low. Based on NRMP Charting Outcomes data, matched orthopedic surgery applicants reported a mean of 16.5 APPs in the pre-transition cohort and 23.8 in the post-transition cohort. Based on our analysis, only 2.0 and 3.7 of those items, respectively, were indexed in PubMed, yielding estimated proportions of 12.1% (pre) and 15.5% (post) (Figure 2). Additionally, further statistics of specific types of published research for each cohort can be found in the Appendices.

## Discussion

Step 1: transition and research productivity 

Our study demonstrated a significant increase in PubMed-indexed research productivity among orthopedic surgery residency applicants following the transition of the USMLE Step 1 examination to pass/fail. Specifically, the mean total number of publications nearly doubled from 1.97 pre-transition to 3.70 post-transition (IRR = 2.13). Similar increases were noted in first-author publications (IRR = 2.28) and in-specialty publications (IRR = 2.32). 

These findings echo the previous literature based on the 2024 NMRP Charting Outcomes, which documented a steep rise in applicant-reported APPs to 23.8, a steep rise from previous APP counts in 2020 and 2022 [6]. This surge in APP numbers after the transition significantly outpaced the expected APP numbers calculated through forecasting analysis by Varieur et. al, which predicted an APP count of 18.4 in 2024 [15]. Our study contributes to the growing body of evidence demonstrating that the Step 1 scoring transition correlates with significantly enhanced research productivity among residency applicants. This phenomenon likely reflects a strategic adaptation by applicants who now emphasize research accomplishments as alternative differentiating factors following the removal of numerical Step 1 scores from the application process. 

NIH funding as an independent predictor 

The NIH funding level remained a significant independent predictor for research productivity among applicants in orthopedic surgery after the Step 1 transition. Regression analysis indicated that students from medical schools receiving higher NIH funding consistently had more total publications, first-author publications, and in-specialty publications than those from medical schools receiving less NIH funding.

These results reflect the pre-existing evidence in the literature of the fact that attending a medical school receiving higher NIH funding is independently correlated with increased research output. Bibliometric analyses have found that matched orthopedic surgery applicants from schools receiving higher NIH funding had significantly more peer-reviewed publications than those from lower-funded schools, even after adjusting for gap years and advanced degree status [16]. These previous findings, in addition to the results from our analysis, support the long-standing “Centers of Excellence” hypothesis, which proposes that public research funding is often concentrated in a small number of institutions [17,18]. This concentration leads to access to more robust infrastructure, mentorship, and exposure to research at these institutions, which allows students at these schools to be more productive. Our study is limited by including only matched applicants, which may underestimate the broader equity gap. Further research should investigate differences in matched and unmatched applicants in all NIH funding tiers. In addition, further research should also investigate intra-tier differences, specifically analyzing the impact of specific mentorship and protected research time. 

Citation lag and time bias

Another notable finding in our study was that despite a drastic increase in publication number after the transition to a pass-fail Step 1, there was not a significant change in citation rates of articles published by medical student applicants in orthopedics. This finding persists even when controlling for years since publication. This finding supports other studies that demonstrated the fact that citation rates per year do not significantly differ between pre- and post-USMLE Step 1 pass/fail cohorts, even with a significant increase in research productivity [6,16].

However, this congruity in citation rates despite a spike in research activity may not necessarily be a reflection of research quality. This finding is likely a reflection of the well-established phenomenon of citation lag, which persists because new articles often do not have enough time to be cited, as may be the case for newer post-transition articles. In addition, the non-linear manner of citation rates, which has been established in recent literature as well, plays a role in the lack of citation numbers in newer articles, particularly in those published after the Step 1 grading transition [16]. Thus, in the evaluation of article quality in a short-term time frame, the use of citation rate as a proxy for article quality in our study may be a limitation.

Proportion of PubMed-indexed publications among APPs

Our study provides a novel contribution by explicitly quantifying the proportion of self-reported APPs that are verified through PubMed indexing. We calculated that approximately 12.1% of APPs reported by orthopedic surgery applicants in the pre-transition period and 15.5% in the post-transition period were PubMed-indexed. Mean APPs increased by 3.65%, while mean PMIDs rose by only 0.85%, indicating that PMIDs are growing at a slower rate than APPs. This indicates that the majority of research activity reported by medical students on ERAS and APP metrics reported in NRMP Charting Outcomes data consists mainly of abstracts, posters, presentations, or non-indexed publications. This finding highlights the limited transparency regarding the composition of APP metrics reported in the NRMP. Students lack clarity on what portion of reported APPs comprises peer-reviewed publications versus abstracts, posters, or presentations.

Our results are consistent with prior research emphasizing similar gaps between reported and verifiable scholarly outputs. For instance, Ngaage et al. (2021) found that approximately 40% of matched orthopedic applicants had no PMIDs [16]. Similarly, neurosurgery literature indicates only 12%-30% of self-reported APPs correspond to PubMed-indexed articles [19]. These findings collectively underscore the need for greater transparency within NRMP reporting systems, enabling applicants, advisors, and program directors to better benchmark and interpret the true scholarly productivity of residency candidates.

Limitations

Despite our robust findings, our study has limitations. First, it relied on publicly available data such as PubMed, potentially omitting relevant non-indexed research activities such as poster presentations, abstracts, case files, book chapters, and non-peer-reviewed publications. Although the exclusion of non-indexed scholarly works might appear as a limitation, it is unlikely to significantly impact our overall findings. Non-indexed publications typically lack consistent documentation, are more challenging to verify, and vary considerably in quality and reporting standards. Second, we did not look at other databases such as Web of Science and Scopus. While we recognize that alternative databases include a more extensive range of journals, PubMed remains the most broadly utilized and consistently applied indexing resource within U.S. medical education research [20]. Third, we acknowledge that differences in research productivity are likely influenced by differences in individual research mentorship quality or students' intrinsic motivations [21]. Fourth, we did not look at variations in medical school curricula. Particularly, the availability of structured summer research programs may substantially affect students' research productivity. Students attending institutions offering dedicated, protected summer research time likely have increased opportunities for scholarly activities, potentially skewing comparative publication counts in their favor compared to peers from institutions lacking such structured opportunities. Additionally, our methodology did not capture publications linked to external institutions or hospitals outside each resident’s primary affiliation. However, because we applied a consistent standardized search protocol across both cohorts, these limitations are likely non-differential, minimizing any potential bias in interpreting our findings. Overall, this study provides an essential framework for understanding the influence of systemic and institutional factors on medical students' research productivity.

## Conclusions

In the absence of a numerical Step 1 score, research productivity has taken on greater importance in the orthopedic residency selection process as evidenced by our significant findings of mean PMIDs having nearly doubled from the pre-transition cohort to the post-transition cohort examined. This shift underscores the need for clearer standards in how research activity is reported and interpreted, particularly as institutions and applicants adapt to evolving expectations. While a rise in pre-residency research productivity has been reported, the growth in mean APPs vs. mean PMIDs before and after the Step 1 transition to pass/fail grading was not compared, as done in this study. This study aims to serve as a quantifiable benchmark of research productivity in terms of publications for those planning to apply to orthopedic surgery residency. Moving forward, efforts to improve transparency in research reporting and to examine how institutional factors influence access to scholarly opportunities will be critical in ensuring that selection criteria remain meaningful, measurable, and fair.

## Appendices

**Table 4:** Distribution of Publication Types

Type	Pre-transition	%	Post-transition	%
Case Reports	117	8.30%	171	6.50%
Clinical Trial	2	0.10%	3	0.10%
Clinical Trial, Phase I	0	0.00%	1	0.00%
Comparative Study	65	4.60%	9	0.30%
Consensus Development Conference	1	0.10%	0	0.00%
Controlled Clinical Trial	1	0.10%	0	0.00%
Editorial	9	0.60%	14	0.50%
Evaluation Study	6	0.40%	3	0.10%
Journal Article	1203	85.50%	2303	87.80%
Meta-Analysis	1	0.10%	17	0.60%
Multicenter Study	0	0.00%	26	1.00%
Observational Study	0	0.00%	5	0.20%
Randomized Controlled Trial	0	0.00%	6	0.20%
Review	1	0.10%	18	0.70%
Systematic Review	1	0.10%	48	1.80%
TOTAL	1407	100.00%	2624	100.00%
